# Establishing a postgraduate programme in orthodontics in the Caribbean: governance, collaborations and challenges, at the University of the West Indies

**DOI:** 10.1038/s41405-026-00424-1

**Published:** 2026-04-03

**Authors:** Trudee Hoyte

**Affiliations:** School of Dentistry, Faculty of Medical Sciences, The University of The West Indies, St. Augustine, Trinidad

**Keywords:** Dental post graduate education, Orthodontics

## Abstract

**Objective:**

To describe the development and implementation of the first postgraduate orthodontic training programme in the English-speaking Caribbean and situate its establishment within international outlines for postgraduate dental education and global health workforce development.

**Methods:**

A descriptive analysis was undertaken of the governance processes, curriculum development and institutional collaborations involved in establishing a postgraduate orthodontic programme at The University of the West Indies (UWI). Programme development incorporated regional workforce needs assessment, stakeholder consultation and benchmarking against internationally recognised competency frameworks, including those of the UK Specialist Advisory Committee and European postgraduate orthodontic education guidelines.

**Results:**

The programme was developed through a multi-stage governance pathway within UWI and in collaboration with the Royal College of Surgeons of Edinburgh. A competency-based curriculum was constructed through structured mapping of programme learning outcomes to international orthodontic training standards. The three-year full-time programme integrates supervised clinical training, theoretical instruction and a research dissertation. Clinical training comprises approximately 24–28 hours weekly with structured workplace-based assessments and competency-based progression. Hybrid teaching models and international faculty support were introduced to address regional staffing limitations.

**Conclusions:**

The establishment of this programme represents a significant milestone in specialist dental education in the Caribbean. By combining international educational standards with regional contextual adaptation, the initiative provides a model for developing sustainable specialist training programmes in small-island and resource-constrained settings.

## Introduction

An essential component of oral health is orthodontic care. This care significantly impacts facial aesthetics, functional occlusion, psychological well-being and quality of life [1, 2]. These associations are well established within oral health related quality of life literature, which recognizes malocclusion as both a clinical and psychosocial condition [1, 2]. In the Caribbean, access to orthodontic treatment remains highly uneven, but there is a growing recognition of the importance of specialized dental services. Patients often face long waiting times, high treatment costs, or the need to travel to another island or country for care because many Caribbean nations lack trained orthodontists.

Over the past two decades, the prevalence of dental anomalies and the burden of malocclusion have increased in the Caribbean [3, 4]. Also, the prevalent malocclusion is bimaxillary protrusion [5]. Despite this, the number of orthodontists remains critically low [6]. Such work force imbalance reflects patterns described in international workforce planning literature, where specialist to population disparities contribute to inequitable access to care and increased cross boarder patient movement.

There are no postgraduate programmes in dentistry in the English, French and Dutch-speaking Caribbean despite the presence of two University of the West Indies (UWI) dental schools in the Caribbean, located in Trinidad and Tobago and Jamaica and one dental school in Haiti and another in Guyana. The absence of local specialist training infrastructure contrasts with global recommendations that advocate for regionally embedded postgraduate programmes to strengthen workforce retention and health system resilience [7]. Consequently, all orthodontists in the region have trained internationally. Training internationally is costly and inflexible [8]. Recent analysis of UK postgraduate dental training pathways highlights barriers to international applicants, including limited flexibility and financial constraints [8]. Such barriers disproportionately affect trainees from small-island developing states. Reliance on overseas education is also associated with professional migration “brain drain”, a phenomenon widely documented in global health workforce literature [9]. Also, there are issues of accessibility and cultural misalignment of orthodontic treatment. This reliance on overseas training also leads to challenges in health system development, workforce planning and retention.

The establishment of a postgraduate programme in Orthodontics at The University of the West Indies (UWI)represents not only a landmark development in the Caribbean region but also a strategic step towards building regional capacity in specialist care, dental education and research. Postgraduate dental education is pivotal for advancing clinical expertise and leadership in oral health in this region. Global health systems research emphasizes that local postgraduate training is a key mechanism for strengthening specialist workforce sustainability and reducing dependence on external systems [7, 9]. Local training programmes also allow curricula to be tailored to regional disease patterns, healthcare infrastructure and patient needs.

Specialist training programmes must therefore be underpinned by structured governance, evidence-based curriculum design, and robust external oversight to foster clinical competence and protect public safety. British standards for assessment integrity and competency-based evaluation, along with the regulatory body role, were deployed throughout the process [10]. Competency-based medical and dental education frameworks emphasize transparent progression, structured assessment and alignment with regulatory standards to ensure patient safety and public protection [10, 11].

After appointing an ambassador to the Caribbean region, the Royal College of Surgeons of Edinburgh (RCSEd) offered to help in developing postgraduate programmes in the region. In response, the University of the West Indies collaborated with the Royal College of Surgeons of Edinburgh to establish a local postgraduate programme in Orthodontics. This paper outlines the issues faced in setting up the first postgraduate programme in the English-speaking Caribbean. It describes the rationale and development process, with the focus on addressing regional needs and ensuring sustainability.

### Setting

Twenty -six islands make up the Caribbean region. Guyana is a country on the northern coast of South America and has historically been identified with the Caribbean and was once part of the British West Indies. For the purpose of this initiative, it was included in the Caribbean region.

The University of the West Indies has five campuses across the Caribbean and one online. Undergraduate dental schools are located only on the Trinidad and Jamaica campuses. The programme was established at the University of the West Indies dental school located in Trinidad. The campus in Trinidad had the longest existing undergraduate programme (35 years) and had the qualified staff and foundational clinical infrastructure in place. A community dental practice model similar to UK models was considered, but the idea was abandoned due to a lack of infrastructure in primary dental facilities located in Trinidad and Tobago [12].

### The unique structure of the faculty of medical sciences

**Fig. 1:**
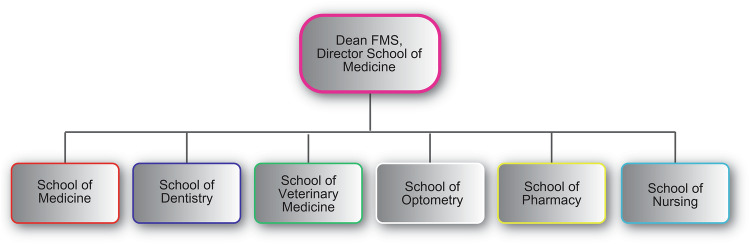
Faculty of Medical Sciences Structure.

Unlike a standalone dental school in other regions. The UWI School of Dentistry is part of the Medical Sciences Faculty. It promotes interdisciplinary collaboration across medicine, dentistry, and other health sciences. Six schools make up the faculty: they are medical, dental, veterinary, optometry, pharmacy and nursing schools. The faculty is run by the faculty dean, who is also the director of the medical school (Fig. 1). Any new programme, especially at the postgraduate level, must pass through several internal and cross-campus committees allowing for multiple levels of institutional review and approval.

### Programme development and governance process

#### Faculty and infrastructure

The University opened a new dental extension in 2020 and, due to the pandemic, was not occupied until 2021 [13]. This new clinical facility was sanctioned for postgraduate training. University grants were used for the development of laboratory staff. Laboratory staff travelled to Cardiff University for mentorship and observership to strengthen local technical capacity.

#### Staffing

Like most dental schools, there is a shortage of teaching staff. There is currently one full-time orthodontist on staff, and another is being actively recruited. The disparity between private practice and academia income has made it very difficult to recruit high-quality lecturers who wish to remain in academia. With only one full-time orthodontist on staff, international faculty were accessed by using the flying faculty model and online teaching through Microsoft Teams and Zoom. These avenues made the establishment of the programme sustainable. This addressed gaps in specialist teaching capacity and lays the groundwork for faculty development and local expertise over time. While a part-time option in the environment where education is delivered is recommended by the General Dental Council(GDC) since it will allow applicants flexibility to work [14], this programme is full-time and will be accepting applicants every other year because of the staffing constraints.

The journey toward establishing the Postgraduate Orthodontic Programme was not only collaborative but methodical. The process began with a needs assessment, followed by the curriculum design.

#### Needs assessment

The Caribbean region has a total population of 45.5 million. The English-speaking Caribbean and Guyana account for approximately 6.8 million people, with Trinidad and Tobago and Jamaica accounting for the most significant portions. This region has 21 orthodontists, 16 residing in Trinidad and Tobago and Jamaica. Most islands do not have an orthodontist. A few Caribbean islands are facilitated with an orthodontist from the three islands travelling to them, but most patients have to travel to another island or the mainland to receive care.

Previously, all orthodontists were trained overseas, which was expensive. As stated previously, training internationally is not flexible and does not allow flexible working for international students [8]. Due to the internet and social media, there is an increasing demand for orthodontic care [15]. There is limited public sector access across the Caribbean, and the majority of orthodontics, when available, is privately funded.

#### Survey

The Royal College ambassador, after contacting dental associations or their equivalent professional organisations in the Caribbean region, sent out a survey to sixteen associations or equivalents. The survey was shared with all registered dentists in that country. The anonymous cross-sectional survey used a self-administered questionnaire design (Survey Planet LLC, Marini Del Ray, CA, USA). The questionnaire consisted of ten questions addressing demographic information, interest in postgraduate training and preferred modes of programme delivery. The survey was conducted to assess training needs and was not a research study. Out of the sixteen associations and equivalent professional organisations, responses were received from three hundred and thirty-seven registered practitioners from thirteen countries. The survey showed a strong interest among dentists for specialty training within the region (72.7% of respondents).

Both the needs assessment and survey laid the foundation for institutional buy-in and will assist in obtaining accreditation.

#### Curriculum design

Curricula are strengthened by explicit competencies and structured assessments that ensure graduates can independently diagnose and manage complex orthodontic conditions. The curriculum was benchmarked against UK Specialist Advisory Committee (SAC) [16] and informed by European postgraduate education guidelines [17]. Contemporary orthodontic education literature emphasizes competency-based training frameworks [11]. Competency-based education (CBE) shifts focus from time-based training to demonstrate outcomes, ensuring graduates can independently diagnose and manage complex orthodontic conditions [11]. In response to contemporary scholarship in dental education, Programme Learning Outcomes (PLOs) were developed prior to curriculum construction. These include knowledge, clinical skills, professional behaviours and research and were expected of graduates at completion of training. The PLOs were structured across six domains: (1) comprehensive diagnosis and treatment planning, (2) biomechanical and appliance therapy; (3) interdisciplinary management, (4) research and critical appraisal, (5) professionalism, ethics and patient safety; (6) leadership within Caribbean oral health systems. The SAC curriculum explicitly links training standards to regulatory oversight, patient safety, quality assurance and structured assessment progression [16].

Rather than directly transplant the UK model, a structured competency- mapping exercise was undertaken with curriculum specialist at UWI. Each SAC competency was mapped against the newly defined PLOs to ensure constructive alignment between intended outcomes, teaching activities and assessment strategies. This process reflects principles described in competency –based dental education literature, which emphasize transparency of outcomes, structured progression and alignment between curriculum components [11].

Its adoption provided an evidence-informed template rather than an ad hoc curriculum design. European postgraduate orthodontic education guidelines were also consulted [17]. European guidelines recommend defined supervision ratios, minimal clinical exposure and integration of research activity within specialist programmes [17]. The programme spans three academic years (minimum 40 weeks per year) [17]. Training is structured to ensure progressive development of clinical autonomy, theoretical knowledge and research competence.

#### Clinical training

Postgraduate students will undertake a minimum of seven supervised clinical sessions per week (each session approximately 3.5 h), equating to 24–28 clinical hours weekly and an estimated 3000–3300 clinical hours over the three- year programme. Each trainee is required to undertake a minimum of 80 comprehensive orthodontic cases, including fixed appliance therapy, interceptive treatment, growth modification, orthognathic multidisciplinary cases and retreatment cases in alignment with European postgraduate orthodontic guidelines [17]. Clinical supervision is delivered at a 1:2 staff-to-student ratio to ensure patient safety and competency- based progression [17] Table 1.

**Table 1:** Structure of the MSc orthodontics programme (3 years full-time).

Component	Description	Approximate distribution
Duration	36 months(full-time)	Continuous enrolment
Clinical training	Supervised management of comprehensive orthodontic cases (adolescent, adult, multidisciplinary, interceptive)	3300 clinical hours over 3 years
Supervisor Ratio	Minimum clinical supervision ratio	1:2 (staff: student) in a clinical session
Didactic teaching	Weekly lectures and seminars (growth & development, biomechanics, treatment planning, evidence-based orthodontics, craniofacial anomalies etc.) Interdisciplinary case conferences Delivered face to face or via zoom/Microsoft teams	4–6 h per week
Interdisciplinary care	Joint case conferences with oral surgery, restorative dentistry and paediatric dentistry	Scheduled sessions each semester
Research component	Original supervised research project, dissertation and viva Aim for publication quality output	Mandatory
Assessment	Workplace- based assessments, case- based discussions, written examinations, OSCE-style assessments, annual review of competence progression	Continuous and summative
External oversight	External examiner input; alignment with standards recognized by the Royal College of Surgeons of Edinburgh	Annual review

#### Theoretical and didactic teaching

Formal didactic teaching comprises 3–6 h per week across seminars, case-based discussions and structured teaching sessions. Theoretical teaching is delivered through:

- Core orthodontic lectures
- Core orthodontic seminars
- Interdisciplinary case conferences
- Research methodology classes (done in conjunction with the faculty of medical sciences)

Across the three years trainees will complete approximately 900-1000 hours of theoretical instruction.

#### Distribution of core topics

The lectures and seminars topics are adapted from the SAC document on generic knowledge, skills and attitudes and orthodontic specialist specific knowledge, skills and attitudes [16] and are distributed longitudinally across the three years and include but not limited to

- Craniofacial growth and development
- Cephalometrics and advance diagnostic imaging
- Biomechanics and appliance systems
- Fixed and removable appliance therapy
- Clear aligner therapy
- Management of impacted teeth
- Interdisciplinary orthodontics (orthognathic surgery, restorative interface)
- Management of bimaxillary protrusion (reflecting regional epidemiology)
- Periodontal considerations
- Cleft and craniofacial anomalies
- Retention and relapse
- Ethics, consent and medicolegal considerations
- Practice management and leadership within Caribbean oral health systems

#### External lecturers and flying faculty

Given local staffing constraints, external lecturers affiliated with the Royal College of Surgeons of Edinburgh and invited academics contribute 90% of theoretical curriculum. Delivery occurs via

- Hybrid online lectures and seminars
- Intensive in-person teaching blocks
- Case discussions and viva style mock examinations

The model supplements local expertise while supporting long-term faculty development.

## Research component

Each postgraduate trainee completes a supervised research dissertation over the three-year programme. Research projects may be clinical, epidemiological, educational in nature and aligned with regional oral health priorities. Completion of the dissertation is mandatory for award of the degree.

Rather than directly transplanting a UK model, contextual adaptation to reflect regional epidemiology, including higher prevalence of bimaxillary protrusion [5, 18], and local healthcare infrastructure constraints were adopted. Literature cautions against uncritical curriculum transfer without contextual modification [19], and therefore regional disease pattern and workforce realities informed final curriculum structure. Postgraduates will maintain logbooks and a competency sign-off documentation [10]. It is recommended that they attend worldwide orthodontic meetings and congresses. The programme will be coordinated by a registered specialist orthodontist with over 18 years’ experience and who is a member of British Orthodontic Society and American Orthodontic Society. The programme will be based on a minimum of 40 weeks per year [17]. The programme spans three years full-time and includes clinical, didactic and research components Table 1.

### Assessment strategy

Assessment is longitudinal and competency-driven. Workplace- based assessments and structured logbooks are consistent with recommendations for improving postgraduate dental assessment reliability and transparency [10]. Therefore, workplace -based assessments (WBAs), structured case-based discussions, OSCE-style assessments and annual review of competence progression have been embedded to ensure both formative and summative evaluation. This multi-modal assessment strategy aligns with recommendation for improving reliability and transparency in postgraduate dental assessment [10] (Table 1). Each postgraduate student undergoes continuous formative assessment through workplace-based assessments (mini-CEX, Case-based discussions, Direct Observation of Procedural Skills (DOPS), Multi-source feedback (MSF), Audit, and observation of teaching), supported by structured logbooks documenting clinical exposure and competency sign-off. Summative assessments include written examinations, structured clinical case presentations, OSCE-style assessments and dissertation defence. An Annual Review of Competence Progression (ARCP-style framework) evaluates trainee advancement against defined competency milestones. External examiner oversight and alignment with standards by the Royal College of Surgeons of Edinburgh provide benchmarking and quality assurance. This structured progression model reflects best practice recommendations for postgraduate dental education assessment [10, 11].

### University governance structure

**Fig. 2:**
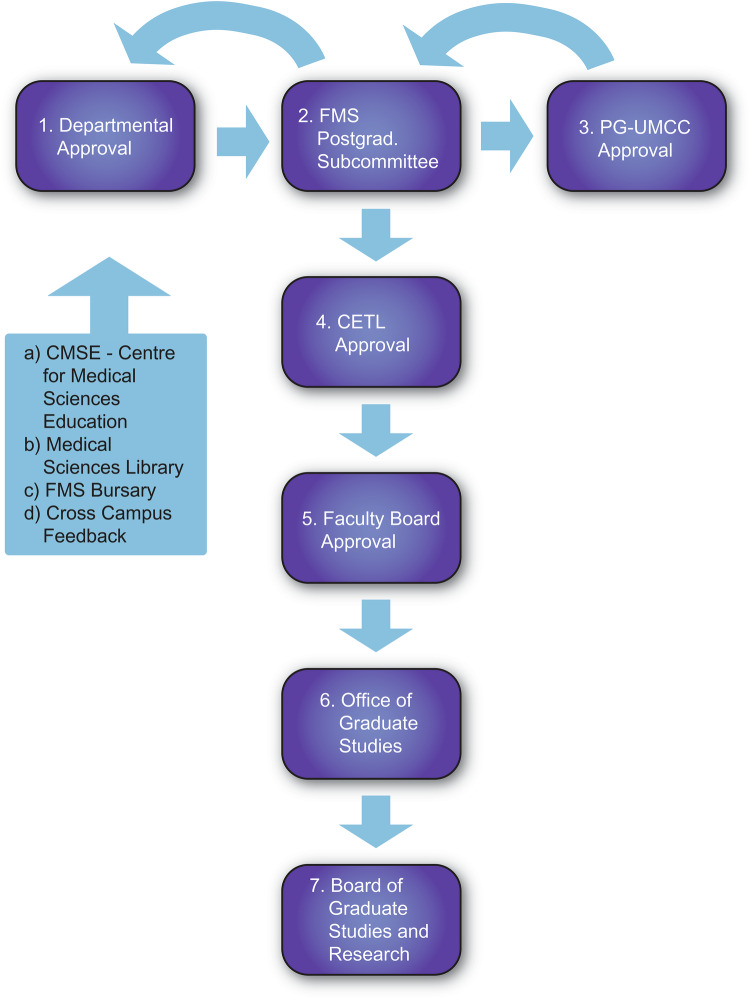
Flowchart of steps for approval of new graduate programmes or courses.

To attain approval, the programme proposal was steered through a series of university governance structures (Fig. 2). This layered governance ensures academic integrity, transparency and alignment with regional workforce needs. It ensures quality assurance and accountability. Each stage involved scrutiny of academic content, staffing, resource allocation, and alignment with the university’s strategic plan. Layered governance and multi-stage academic scrutiny are recognized mechanisms for ensuring programme integrity, particularly within clinical disciplines where patient safety and supervision ratios directly influence training quality. Each stage evaluated staffing, infrastructure and financial sustainability.

This governance pathway also reflects broader higher education quality assurance frameworks, which emphasize transparency, accountability and strategic alignment [20]. Within clinical education, such layered scrutiny functions not only as administrative oversight but as educational safeguard to ensure that supervision ratios, infrastructure readiness and patient access meet standards necessary for safe specialist training.

### Stakeholder input

Stakeholders from across the UWI multi-campus system (Trinidad, Jamaica, Barbados, Five Islands and the Open Campus) read the proposal, which included the programme description, rationale, business motivation, demand, marketing and advertising, access and support, course of study, faculty, regulations and assessment procedures, quality assurance, curriculum, and budget. The proposal underwent a rigorous academic review and modification based on feedback. This ensured alignment with broader regional goals. The medical sciences library also prepared an impact statement. The programme had to be sustainable; therefore, orthodontic fees for services had to be charged. Postgraduate students also had to pay a tuition fee. Therefore, the university bursary had to review the prepared budget in the proposal to confirm it was profitable and achievable.

Because the curriculum was adopted from the UK, SAC/GDC, the Centre for Medical Sciences Education (CMSE) and faculty curriculum steps were bypassed.

### Departmental approval

The school reviewed the proposal and gave stakeholder input. They made edits and comments before approval and moving on to the next phase.

### Graduate studies and research Committee

The proposal was then sent to the faculty graduate studies and research committee to evaluate for academic merit and agreement with postgraduate standards. Once passed by this committee. It was sent to the Centre for Excellence in Teaching and learning (CETL).

### CETL

At CETL, the curriculum was analyzed in detail by a curriculum specialist. After this, the proposal was presented to the faculty board (all schools were present).

### Board of graduate studies and research

The proposal was then scrutinized by the university board of graduate studies and research.

Approval from this committee is essential to align the programme with institutional polices, academic standards and quality assurance processes.

### Finalising curriculum design

Submitting the proposal to the campus finance and general purpose committee (CFGPC) was the final board where the finances and overall programme were discussed. The programme received CFGPC approval in April 2025 and the first cohort of postgraduate students was accepted on 15th September 2025.

### International collaborations

The Royal College provided advisory support. International academic partnerships have been described in the literature as effective mechanisms for specialist capacity- building when structured around sustainability rather than dependency [5, 9]. Some members of the Royal College will be assisting as either flying faculty and hybrid digital delivery. Hybrid delivery models have increasingly been integrated into postgraduate dental education following the COVID-19 pandemic, demonstrating feasibility for international collaboration without physical reaction [13].The programme intends to seek accreditation from the RCSEd. Alignment with RCSEd standards will enable examination eligibility and international recognition. Accreditation alignment ensures external benchmarking, a key feature of internationally recognized specialist training frameworks [16, 17].There will be an opportunity for postgraduate students to sit their specialty exams once accreditation from RCSEd is received. This would ensure credibility and mobility of graduates, and the curriculum would reflect international benchmarks. Achieving this requires careful mapping of competencies, clinical hours and evaluations.

Because the programme had to be sustainable, donations were received from American Orthodontics. The donations included fixed appliance hardware, accessories, and equipment to cover the first two years of the programme. Cardiff University offered training to the laboratory technician.

There has long been an orientation to overseas postgraduate education. Building trust in the UWI programme in the early years was essential to overcoming scepticism about quality and recognition.

### Challenges encountered

Dentistry differs from other disciplines in its requirements for regulatory oversight, clinical infrastructure and accreditation. Securing approval at departmental, faculty and university-wide levels was a prolonged process. Launching this postgraduate programme was resource-intensive. Traditional university funds were insufficient to cover initial costs. A balance needed to be struck between academic rigour and financial sustainability.

Attracting and retaining highly trained specialists was and is one of the most significant barriers. There is a vast disparity between private practice earnings and earnings in academia. Therefore, many potential candidates are hesitant to make the transition into academia without adequate incentives.

### Sustainability and staffing solutions

Initial establishment required infrastructure readiness, laboratory development and material acquisition. Industry donations (American Orthodontics) supported early equipment needs, while financial modelling incorporated tuition income and supervised clinical service revenue. Faculty recruitment remains a challenge due to disparities between private practice and academic remuneration. To mitigate this, the programme integrates hybrid teaching models (in person and online) and international collaboration (flying faculty model) while pursuing long-term faculty development. There is active recruitment of another specialist orthodontist and the long- term aim to train graduates into academic roles. The revenue model combines tuition and supervised clinical service income.

### Programme admissions

Applicants must possess a recognized dental degree (BDS or DDS) with a minimum of 1 year dental experience post graduating. Possession of Membership of the Faculty of Dentistry (MFDS) is now mandatory. A maximum of 2 students per intake (due to staffing and caseload considerations). Intake will be in alternate years. Annual tuition fee (250,000TT for nationals and 45,000USD for non- nationals). Clinical services revenue will support sustainability of the programme.

## Discussion

This paper contributes to literature on postgraduate dental education in resource-constrained settings by presenting a goveranance-informed competency-mapped model for specialist programme establishment in a small-island developing region. The initiative represents not merely programme creation but a structured educational reform grounded in competency- based education. The programme operationalises principles described in global workforce and educational transformation literature, which emphasizes locally embedded postgraduate training as essential for sustainable specialist care [7, 9, 20].

The creation of a postgraduate programme in this region of the world could not have happened without collaboration and support from international institutions such as the Royal College and other tertiary institutions, such as Cardiff University.

The involvement of the Royal College of Surgeons of Edinburgh, an internationally recognised institution, gave structure and credibility. Alignment with internationally recognized competency frameworks [16, 17] allowed external benchmarking while preserving the context in which the curriculum was adapted. The calls in the education literature to avoid uncritical curriculum transplantation is reflected in the balance sought between global standards and regional relevance [20].

The University of the West Indies will guarantee accessibility in the region and relevance. The gaps in specialist teaching capacity were addressed by the flying faculty and the use of modern technologies like Microsoft Teams and Zoom. This will also aid in laying the groundwork for faculty development and local expertise over time.

The establishment of the first postgraduate programme in orthodontics poses unique administrative and academic challenges. These involve recruiting qualified faculty, pursuing accreditation from internationally recognized bodies such as The RCSEd, ensuring access to patient caseloads and providing cost-effective postgraduate education.

The importance of having a competency based curricula cannot be underscored [11]. By mapping programme learning outcomes to SAC competencies [16], integrating workplace-based assessment strategies [10] the programme demonstrates constructive alignment between PLO, teaching methods and assessment strategies. This alignment is central to contemporary teaching and learning in health profession education [11]. Using the orthodontic specialty curriculum from the GDC, UWI was able to outline standards for training their postgraduate students link it to patient safety, quality evaluation and continuous improvement through research and assessment alignment [16].

While governance structures are often looked at as administrative hurdles, in this context the multi-tiered approval process functioned as an embedded quality assurance mechanism. In clinical education, where supervision ratios, infrastructure readiness and patient access directly influence patient safety, such scrutiny represents an educational safeguard rather than bureaucratic delay.

It is the first structured postgraduate programme in the English-speaking Caribbean and has adapted the UK regulatory standards [16] and European guidelines [17] to a small-island developing region without compromising competency standards. In doing so it contributes to literature on specialist capacity building in resource constrained settings and offers a governance and curriculum framework for similar underserved regions [21].

Global workforce literature underscores the importance of locally embedded postgraduate training for retention and sustainability [9, 21]. It operationalizes those principles within dentistry.

The literature frequently examines medicine and nursing migration, specialist dental workforce sustainability remains comparatively underexplored. This initiative provides a case example of how postgraduate dental education can serve as a structural intervention against specialist migration in small-island developing states. It has demonstrated how international collaboration enables specialist capacity building.

This work extends existing international orthodontic curriculum standards by demonstrating a structured competency- mapping process that balances global regulatory alignment with epidemiological specificity. Rather than curriculum transplantation, the programme adopted a contextual adaptation model informed by regional malocclusion patterns, healthcare infrastructure constraints and workforce realities. This approach can be a template for other small -island or low -resource regions seeking international benchmarking without educational dependency.

Additionally, ensuring stakeholder engagement across a diverse Caribbean region while ensuring financial sustainability added a layer of complexity to programme establishment.

Although developed within the Caribbean, the model described here may be transfereable to other underserved regions characterized by small populations, limited faculty capacity and reliance on overseas specialist training. The integration of hybrid teaching, flying faculty models and structured competency mapping offers a scalable approach for emerging postgraduate programmes globally.

## Conclusion

This programme can be a model for setting postgraduate programmes in underserved regions, aiming to develop their training capacity for specialists once it has continued investment, regional cooperation and policy support.

## Data Availability

All data is available from the corresponding author.
